# Lysophosphatidic acid acyltransferase 3 tunes the membrane status of germ cells by incorporating docosahexaenoic acid during spermatogenesis

**DOI:** 10.1074/jbc.M117.791277

**Published:** 2017-06-03

**Authors:** Yoshiko Iizuka-Hishikawa, Daisuke Hishikawa, Junko Sasaki, Keiyo Takubo, Motohito Goto, Katsuyuki Nagata, Hiroki Nakanishi, Hideo Shindou, Tadashi Okamura, Chizuru Ito, Kiyotaka Toshimori, Takehiko Sasaki, Takao Shimizu

**Affiliations:** From the a Department of Lipid Signaling, National Center for Global Health and Medicine, Shinjuku-ku, Tokyo 162-8655,; the b Department of Medical Biology, Akita University Graduate School of Medicine, Akita 010-8543,; the Departments of c Stem Cell Biology and; e Laboratory Animal Medicine, National Center for Global Health and Medicine, Shinjuku-ku, Tokyo 162-8655,; the f Research Center for Biosignal, Akita University, Akita 010-8543,; the h Section of Animal Models, Department of Infectious Diseases, National Center for Global Health and Medicine, Shinjuku-ku, Tokyo 162-8655,; the i Department of Reproductive Biology and Medicine, Graduate School of Medicine, and; j Future Medicine Research Center, Chiba University, Chiba 260-8670,; the Departments of g Lipid Science and; k Lipidomics, Graduate School of Medicine, University of Tokyo, Bunkyo-ku, Tokyo 113-0033, and; the d Agency for Medical Research and Development (AMED)–Core Research for Evolution Science and Technology (CREST), Chiyoda-ku, Tokyo 100-0004, Japan

**Keywords:** glycerophospholipid, membrane enzyme, phospholipid metabolism, polyunsaturated fatty acid (PUFA), spermatogenesis

## Abstract

Docosahexaenoic acid (DHA) is one of the essential ω-3 polyunsaturated fatty acids with a wide range of physiological roles important for human health. For example, DHA renders cell membranes more flexible and is therefore important for cellular function, but information on the mechanisms that control DHA levels in membranes is limited. Specifically, it is unclear which factors determine DHA incorporation into cell membranes and how DHA exerts biological effects. We found that lysophosphatidic acid acyltransferase 3 (LPAAT3) is required for producing DHA-containing phospholipids in various tissues, such as the testes and retina. In this study, we report that LPAAT3-KO mice display severe male infertility with abnormal sperm morphology. During germ cell differentiation, the expression of LPAAT3 was induced, and germ cells obtained more DHA-containing phospholipids. Loss of LPAAT3 caused drastic reduction of DHA-containing phospholipids in spermatids that led to excess cytoplasm around its head, which is normally removed by surrounding Sertoli cells via endocytosis at the final stage of spermatogenesis. *In vitro* liposome filtration assay raised the possibility that DHA in phospholipids promotes membrane deformation that is required for the rapid endocytosis. These data suggest that decreased membrane flexibility in LPAAT3-KO sperm impaired the efficient removal of sperm content through endocytosis. We conclude that LPAAT3-mediated enrichment of cell membranes with DHA-containing phospholipids endows these membranes with physicochemical properties needed for normal cellular processes, as exemplified by spermatogenesis.

## Introduction

In the body, fatty acids are mainly stored in membrane phospholipids and neutral lipids and act as the structural components of the cell membrane, an energy source, and precursors of bioactive lipid mediators (1). Because the physical and chemical properties of fatty acids differ depending on the carbon chain length and degree of unsaturation, the fatty acid composition of phospholipids affects membrane fluidity, flexibility, fusion, fission, and curvature (2, 3). Polyunsaturated fatty acids (PUFAs) render the membrane flexible, whereas saturated or *trans*-fatty acids rigidify it (2, 4, 5). Although the fatty acid composition of membrane phospholipids varies among tissues and they are thought to be important for the cellular functions, little is known about the influence of physical properties of membranes *in vivo* (6).

Docosahexaenoic acid (DHA,^4^ 22:6) is one of the ω-3 PUFAs with 22 carbon chains and 6 double bonds. Because mammals cannot synthesize ω-3 fatty acids (7), we need to obtain them from marine dietary sources (8). The nutritional importance of ω-3 PUFAs was first proposed in 1932 (9) and attracted more attention by studies in the late 1970s, which showed a correlation between low incidence of myocardial infarction and high dietary intake of ω-3 PUFAs (10, 11). Identification of ω-3 fatty acid-derived pro-resolving mediators (protectins, resolvins, and maresins) (12) also expands our understanding of the roles of ω-3 PUFAs besides being energy sources and membrane phospholipid components. To date, numerous studies have reported the beneficial effects of ω-3 PUFAs on health, and DHA deficiency is associated with hyperlipidemia, cardiovascular disease, cognitive dysfunction, and retinal degeneration (8). In addition, ω-3 PUFAs (especially DHA) appear important for sperm formation and male fertility in mammals, whereas the increased intake of saturated or *trans*-fatty acids is reported to correlate with a decrease in male reproductive ability in humans in recent years and to be a risk factor for male infertility (13–18). Together with the fact that mammalian sperm possesses a high amount of PUFA, including DHA (19), it is possible that highly fluid and flexible membrane is required for sperm formation and functions. Recent gene knock-out strategies as well as analyses of human genetic disorders have unveiled several important molecules involved in the uptake and trafficking of DHA (20–22); however, the mechanism of how lipid profiles affect the male reproductive system is not well understood.

Here, we found that lysophosphatidic acid acyltransferase 3 (LPAAT3), also known as 1-acylglycerol-3-phosphate *O*-acyltransferase 3 (AGPAT3), is the critical enzyme for producing DHA-containing membrane phospholipids in various tissues, such as the testes and retinas (also refer to the accompanying paper by Shindou *et al.* (49)). LPAAT3 knock-out (KO) mice showed severe male infertility due to the defect in the final step of spermatogenesis as a consequence of a specific and dramatic decrease in DHA-containing phospholipids. Thus, this study represents the insight into how DHA is required in the body and provides a new mammalian context to study the DHA-related health and diseases.

## Results

### LPAAT3 is expressed in mature spermatids and Leydig cells in the testes

**Figure 1.**
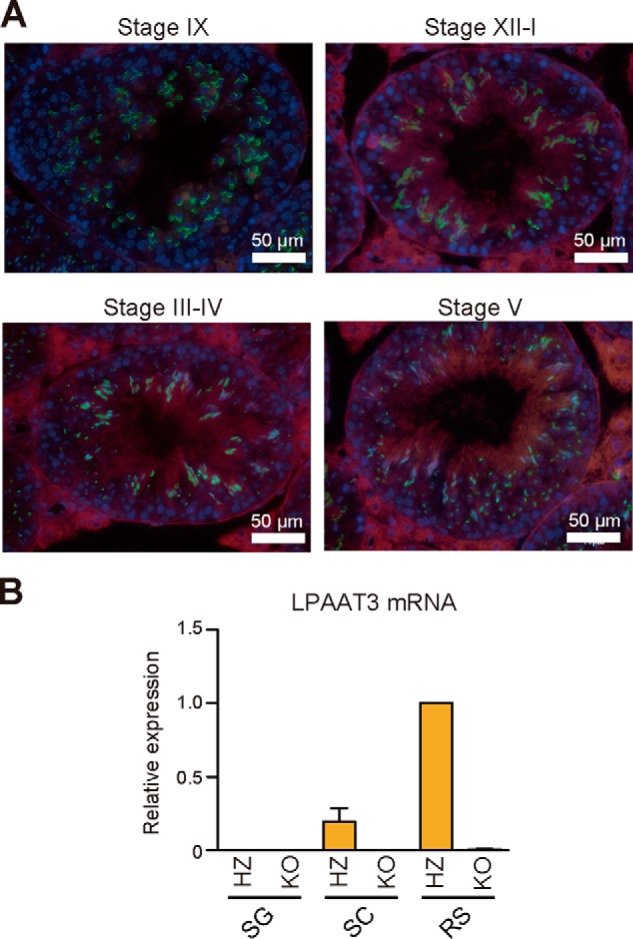
**LPAAT3 expression in testes.** *A,* representative immunohistochemical images of mouse testes. LPAAT3 is in *red*; acrosome is in *green*, and nucleus is in *blue*. Acrosome and nuclear staining was used to stage seminiferous tubules. *B,* relative mRNA expression of *LPAAT3* in differentiating germ cells from HZ and KO mice. *LPAAT3* mRNA expression was normalized to *GAPDH. SG*, spermatogonia; *SC*, spermatocyte; *RS*, round spermatid (see also supplemental Fig. S2, *A–C*). *Error bars* are S.D. (*n* = 3).

We previously reported that LPAAT3 is an enzyme producing PUFA-containing phosphatidic acid (PA). LPAAT3 is expressed predominantly high in the testes, and it is induced during sexual maturation (23, 24). To determine which cells of the testes express LPAAT3, immunohistochemical analyses were performed. LPAAT3 was expressed mainly in spermatids and Leydig cells (Fig. 1*A*; supplemental Fig. S1). In spermatids, LPAAT3 expression was detected in step 11 spermatids, and the levels increased during differentiation (S11 to S16 in supplemental Fig. S1). *LPAAT3* mRNA expression in germ cells was also investigated. Spermatogonia, spermatocytes, round spermatids, and elongated spermatids were isolated from mouse testes (supplemental Fig. S2, *A–C*). A recent study (25) demonstrated the induction of *LPAAT3* mRNA expression in an *in vitro* model of differentiation from spermatogonial stem cells to the spermatocyte. Consistent with this *in vitro* model, the level of *LPAAT3* mRNA was strongly induced from spermatogonia to the spermatocyte. Additionally, we found that *LPAAT3* mRNA expression was further induced from spermatocyte to round spermatids (Fig. 1*B*). These results suggest that LPAAT3 might play a role in sperm differentiation and/or functions.

### LPAAT3 is required for the production of DHA-containing phospholipids in the testes

**Figure 2.**
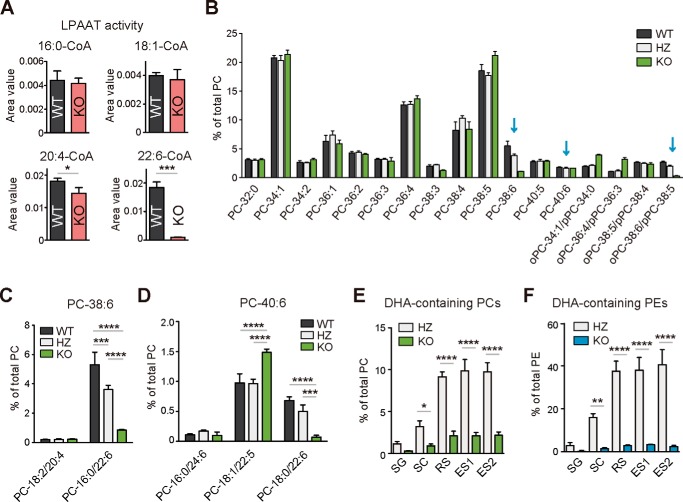
**LPAAT3 is required for the production of DHA-containing phospholipids in testes.** *A,* LPAAT activity in the microsomal fraction of testes in WT and KO mice. Mixed acyl-CoA substrates were used in a single assay. *, *p* < 0.05; ***, *p* < 0.001; unpaired *t* test. *B,* PC composition in the testes. *Arrows* indicate the putative DHA-containing species. *C* and *D,* fatty acid composition of PC-38:6 (*C*) and PC-40:6 (*D*), shown in *B. E* and *F,* percentage of DHA-containing PCs (*E*) and PEs (*F*) in germ cells from HZ and KO mice. *ES*, elongated spermatid (see also supplemental Fig. S2, *A–C*). *C–F,* *, *p* < 0.05; **, *p* < 0.01; ***, *p* < 0.001; ****, *p* < 0.0001; two-way ANOVA, Bonferroni's multiple comparison test. *A–F, error bars* are S.D. (*n* = 3).

To study the role of LPAAT3 *in vivo*, we generated global LPAAT3 knock-out (KO) mice (supplemental Fig. S3, *A–C*). Previous *in vitro* studies demonstrated that LPAAT3 is an enzyme that produces PA containing PUFA, such as arachidonic acid (20:4) and DHA (23, 24, 26, 32). Thus, we first examined the impact of LPAAT3 depletion on the LPAAT activities in wild-type (WT) and KO testes. Consistent with its substrate preference *in vitro*, the LPAAT activities on 20:4-CoA (arachidonoyl-CoA) and 22:6-CoA (DHA-CoA) in the testes were significantly decreased in LPAAT3-KO mice (Fig. 2*A*). The dramatic decrease of the enzyme activity toward DHA-CoA was seen (Fig. 2*A*). Thus, LPAAT3 appears to be the primary enzyme incorporating DHA into LPA *in vivo*.

We then examined how the decreased activity of DHA incorporation into LPA brings change to the composition of membrane phospholipids. In agreement with the observed LPAAT activities, PA-38:6 and 1-*O*-alkyl-2-acyl PA-38:6, which are presumed to contain DHA, were decreased in LPAAT3-KO testes (supplemental Fig. S4*A*). Because PA is a common intermediate for phospholipids, we examined the effect of LPAAT3 deficiency on the fatty acid composition of three major membrane phospholipids in the testes: phosphatidylcholine (PC), phosphatidylethanolamine (PE), and phosphatidylserine (PS). Similar to the results obtained for PA, phospholipids presumably containing DHA were decreased in LPAAT3-KO testes (Fig. 2*B*; supplemental Fig. S4, *B* and *C*). To confirm whether DHA-containing PCs were really decreased, the individual species of PC-38:6 (PC-18:2/20:4 and PC-16:0/22:6) and PC-40:6 (PC-16:0/24:6, PC-18:1/22:5, and PC-18:0/22:6) were analyzed. It is clear that DHA-containing species were selectively decreased in LPAAT3-KO mice (Fig. 2, *C* and *D*).

Considering that LPAAT3 expression is increased during spermatogenesis (Fig. 1, *A* and *B*), alterations of phospholipid compositions in this step in LPAAT3-KO mice and heterozygote (HZ) mice were also compared. Highly unsaturated fatty acids, such as DHA- and docosapentaenoic acid (DPA, 22:5)-containing PC and PE species, were increased during spermatogenesis and displayed the most enrichment in haploid cells (RS, ES1, and ES2 in supplemental Fig. S5, *A* and *B*). However, the increase in DHA-containing species was strongly suppressed in LPAAT3-KO germ cells (Fig. 2, *E* and *F*). These results indicate that LPAAT3 is required to enrich DHA-containing membrane phospholipids during spermatogenesis.

### Lack of LPAAT3 leads to male infertility

**Figure 3.**
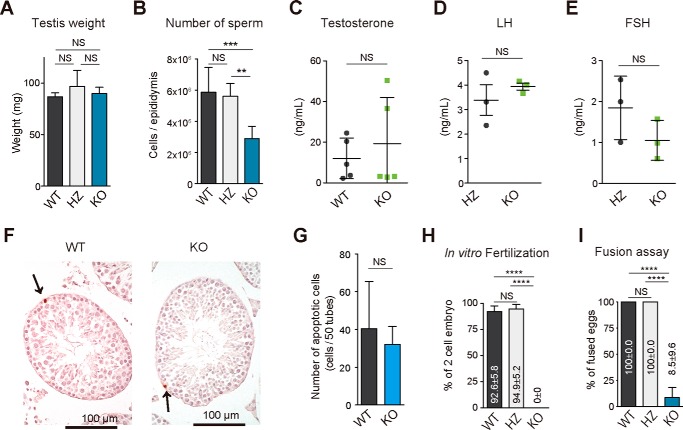
**Loss of LPAAT3 leads to male infertility.** *A* and *B,* testis weight (*A*) and the number of epididymal sperm (*B*) of WT, HZ, and KO mice. *C–E,* levels of testosterone (*C*), LH (*D*), and FSH (*E*) in serum. *Error bars* are S.D. (*C*, *n* = 5; *D* and *E, n* = 3). *F,* representative images of TUNEL staining of LPAAT3 WT and KO testis. *Arrows* indicate the TUNEL-positive apoptotic germ cells. *G,* numbers of apoptotic cells per 50 seminiferous tubules. The sum of TUNEL-positive germ cells in 50 different tubules from one section was indicated. *Error bars* are S.D. (*n* = 3). *H* and *I,* ratio of successful IVF (*H*) and sperm-egg fusion (*I*). *Error bars* are S.D. (*n* = 3). The average values and S.D. are shown. *A, B, H,* and *I,* **, *p* < 0.01; ***, *p* < 0.001; ****, *p* < 0.0001; *NS* indicates not significant; one-way ANOVA, Bonferroni's multiple comparison test. *C–E* and *G, NS* indicates not significant; unpaired *t* test.

We next investigated the roles of LPAAT3 *in vivo*. Notably, we could not obtain any pups from male LPAAT3-KO mice by natural mating, suggesting that LPAAT3-KO male mice were infertile. To assess the cause of male infertility, the testis weight, the number of sperm, and the level of male reproductive hormones, such as testosterone, luteinizing hormone (LH), and follicle-stimulating hormone (FSH), were analyzed. The number of sperm from LPAAT3-KO caudal epididymis was half of those from WT and HZ epididymis, whereas other factors were comparable with control mice (Fig. 3, *A–E*). The apoptotic cells were not increased in the LPAAT3-KO testes, suggesting that apoptosis of germ cells is not the cause of a decreased number of sperm (Fig. 3, *F* and *G*). We further evaluated fertility of LPAAT3-KO sperm by *in vitro* fertilization (IVF) and sperm-oocyte fusion assays. LPAAT3 WT and HZ sperm fertilized more than 90% of WT eggs 24 h after insemination, whereas sperm from LPAAT3-KO mice fertilized no eggs (Fig. 3*H*). In sperm-oocyte fusion assays, the rate of LPAAT3-KO sperm fusing with WT eggs lacking the zona pellucida were extremely low (<10%) (Fig. 3*I*). Cumulatively, these results indicate that the cause of male infertility in LPAAT3-KO mice was the incompetence of sperm rather than the decreased number of sperm.

### LPAAT3-KO sperm display morphological abnormalities

**Figure 4.**
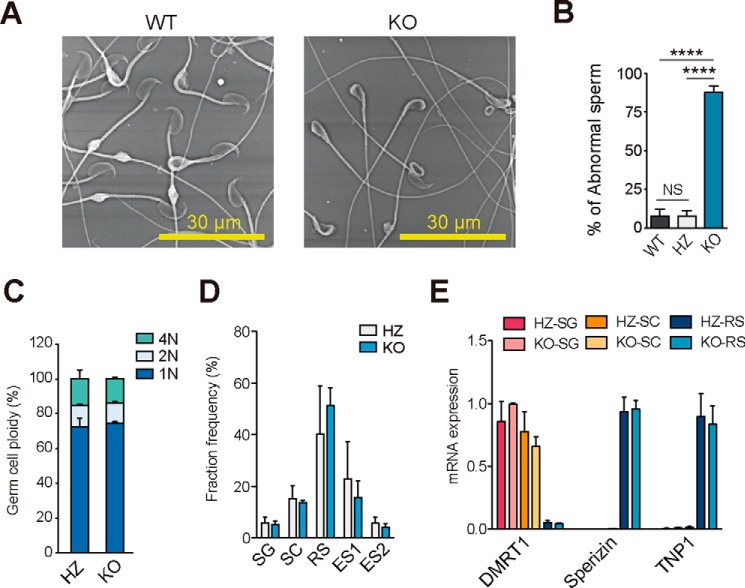
**LPAAT3-KO sperm display morphological abnormalities.** *A,* morphological analysis using low vacuum SEM. *B,* ratio of abnormal sperm from WT HZ and KO epididymis. *C,* ploidy of testicular germ cells from HZ and KO mice. *D,* fraction frequency of isolated germ cells. *E,* mRNA expression of differentiation markers in germ cells. *DMRT1*, Doublesex-related transcription factor 1; *TNP1*, transition protein 1. *Error bars* are S.D. (*n* = 3). *B,* ****, *p* < 0.0001, *NS* indicates not significant; one-way ANOVA, Bonferroni's multiple comparison test. *C–E,* there are no significant differences between HZ and KO mice; one-way (*C* and *D*) or two-way (*E*) ANOVA, Bonferroni's multiple comparison test.

To determine the cause of sperm dysfunctions, we analyzed sperm morphology and testis histology in detail. Remarkably, as many as 90% of the KO sperm displayed heads that bent backwards (Fig. 4, *A* and *B*). As there is no significant difference in ploidy, fraction frequency of germ cells, or the expression of representative differentiation markers between HZ and KO mice, LPAAT3-KO germ cells appeared to differentiate normally into haploid cells (Fig. 4, *C–E*).

**Figure 5.**
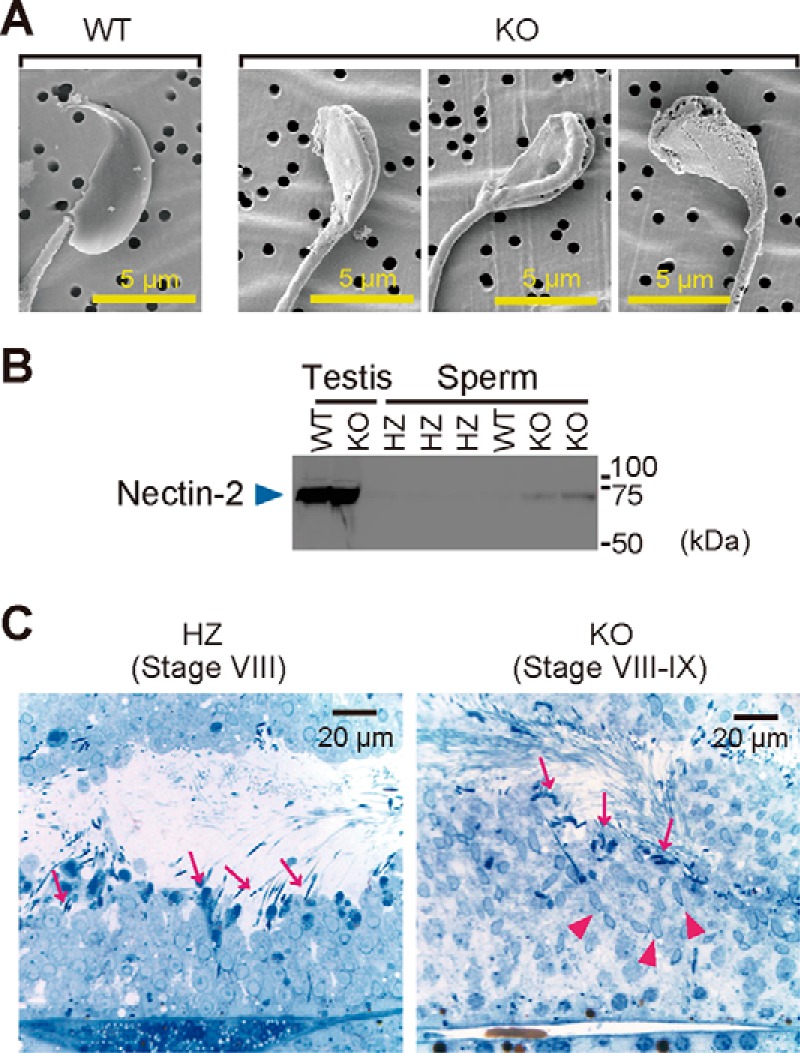
**Delayed sperm release in LPAAT3-KO mice.** *A,* representative SEM images of sperm heads. *B,* expression of nectin-2 in testes and epididymal sperm. *C,* toluidine blue staining of stage VIII–IX seminiferous tubules of HZ and KO testis. *Arrows* and *arrowheads* indicate step 16 and step 9 spermatids, respectively. Normally, step 9 and step 16 spermatids do not coexist in the same stage of seminiferous tubules (see also supplemental Fig. S1). Delayed mature (step 16) sperm release in LPAAT3-KO testes prevent us from discriminating between stage VIII and stage IX.

Analyses using scanning electron microscopy (SEM) revealed that, unlike WT sperm, membranous structures wrapped around the LPAAT3-KO sperm head (Fig. 5*A*). The substances around the LPAAT3-KO sperm head appear to be plasma membrane of Sertoli cells because nectin-2, a cell adhesion molecule expressed only in the Sertoli cells, was detected in the epididymal sperm of LPAAT3-KO but not HZ or WT mice (Fig. 5*B*). This suggests sperm–Sertoli cell junctions were not successfully removed when the sperm were released into seminiferous tubules. Coexistence of step 9 and step 16 spermatids (refer to supplemental Fig. S1) in the same stage of seminiferous tubules in LPAAT3-KO testis, which do not coexist normally (Fig. 5*C*), also indicated that detachment of sperm–Sertoli cell junctions were perturbed in LPAAT3-KO mice. Taken together, these results suggest that LPAAT3-KO mice failed to release mature sperm competent for normal fertilization.

### DHA-containing flexible membrane is required for sperm maturation

**Figure 6.**
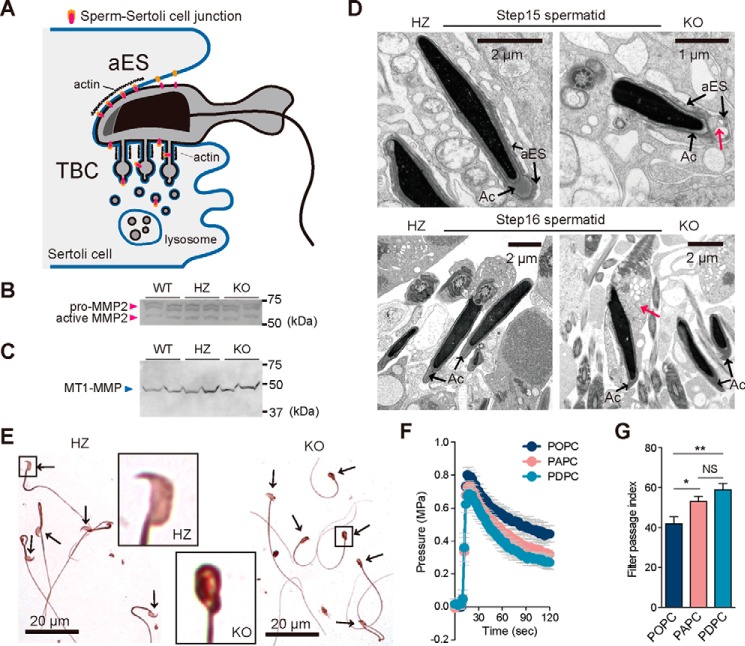
**DHA-containing phospholipid-enriched flexible membrane is required for sperm maturation.** *A,* schematic image of aES and TBC. Both protease-mediated degradation at aES and endocytosis-mediated lysosomal degradation through TBC are necessary to remove sperm–Sertoli cell junctions. *B* and *C,* MMP2 (*B*) and MT1-MMP (*C*) expression in testicular cytosolic and microsomal fraction were analyzed by Western blotting. *Arrowheads* indicate the band of pro-MMP2 (72 kDa), proteolytically activated MMP2 (66 kDa), and MT1-MMP (66 kDa). *D,* TEM images of step 15 and 16 spermatids in HZ and KO testes. *Magenta arrows* indicate excess cytoplasm in LPAAT3-KO sperm. *Black arrows* indicate acrosome (*Ac*) and aES. *E,* immunocytochemical staining of sperm cytoplasm using anti-ubiquitin antibody. *Arrows* indicate the sperm heads. *Boxed areas* were magnified. *F* and *G,* results of liposome filtration assay. Pressure change (*F*) and filter passage index (*G*) for each liposome. POPC (PC-16:0/18:1); PAPC (PC-16:0/20:4); PDPC (PC-16:0/22:6). *Error bars* are S.D. (three technical replicates). Similar results were obtained in five independent experiments. *, *p* < 0.05; **, *p* < 0.01; *NS* indicates not significant; one-way ANOVA, Bonferroni's multiple comparison test.

We finally approached the mechanisms of the delayed sperm release (spermiation) in LPAAT3-KO testes. Prior to sperm release, the sperm–Sertoli cell junction is removed by the coordinated action of two different machineries (27). One is the cleavage of cell-adhesion molecules at apical ectoplasmic specialization (aES) by proteases (28), and the other is the endocytosis-mediated lysosomal degradation through tubulobulbar complexes (TBC) (Fig. 6*A*) (29). Disruption of those machineries is known to lead the delay of spermiation (30). The aES is the testis-specific adherence junction anchoring the elongated sperm until release. Just before spermiation, aES is removed by several proteases, such as matrix metalloproteinases (MMPs) (30). However, the expression of MMP-2 and membrane type-1 MMP (MT1-MMP) was not altered in LPAAT3-KO testes (Fig. 6, *B* and *C*). Also, we could not find the aES around the released LPAAT3-KO sperm (Fig. 6*D*), suggesting the aES is normally removed in LPAAT3-KO sperm. Thus, we next focused on the TBC. The TBC is composed of narrow actin-lined tubules (∼50 nm in diameter), bulbous portions, and clathrin-coated pits (Fig. 6*A*). At the terminal end of the tubule, there are numerous double-membrane vesicles produced by clathrin-mediated endocytosis. These vesicles are subjected to lysosomal degradation of sperm components and spermatid–Sertoli cell junctions. Therefore, if the degradation of sperm–Sertoli cell junctions by TBC is disrupted in LPAAT3-KO mice, elimination of sperm cytoplasm should also be disturbed. Accordingly, we determined whether or not the elimination of cytoplasm was impaired in LPAAT3-KO sperm. Transmitted electron microscopy (TEM) analyses suggested that the excess cytoplasm was retained around the head of released LPAAT3-KO sperm (*magenta arrows* in Fig. 6*D*). Consistent with this observation, ubiquitin, a marker of cytoplasm, was more enriched in the LPAAT3-KO sperm heads compared with WT sperm (Fig. 6*E*). These results strongly suggest that the function of TBC is impaired in LPAAT3-KO mice.

We examined the potential relationship between dysfunction of TBC and changes in phospholipid composition, especially the decrease in DHA-containing phospholipids in LPAAT3-KO mice. DHA-enriched membrane is recently reported to promote the endocytosis by facilitating the membrane deformation and fission (31). This *in vitro* study prompted us to hypothesize that rapid double-membrane-based endocytosis at the TBC requires the DHA-enriched flexible membrane. Because Pinot *et al.* (31) compared the liposome deformation and fission between oleic acid (18:1)- and DHA-containing phospholipids, we further examined whether or not the degree of unsaturation of the fatty acid in phospholipids affected those using the liposome filtration assay (supplemental Fig. S6). We employed the filtration assay system, as no method is currently available for functional assays of TBC. We compared the ease of passage (filter passage index) of liposomes composed of different kinds of PCs (PC-16:0/18:1, PC-16:0/20:4, and PC-16:0/22:6). As a result, the filter passage index of DHA-containing PC liposomes (PC-16:0/22:6) was significant and tended to be higher than those of PC-16:0/18:1 and PC-16:0/20:4 liposomes, respectively (Fig. 6, *F* and *G*). From these results, we propose that LPAAT3-mediated production of DHA-enriched flexible membrane is required for the normal spermiation.

## Discussion

It has been known that ω-3 fatty acids, including DHA, have clinical and nutritional effects different from other PUFAs (8). However, the regulatory mechanisms of DHA incorporation into the cell membrane remain unknown thus limiting our understanding of the role of DHA *in vivo*. Here, we demonstrated that LPAAT3 is the essential enzyme to produce the DHA-containing phospholipids in the testes. This was consistent with our recent hypothesis that the amount of DHA in cell membranes is predominantly regulated by LPAATs, enzymes required for *cha* phospholipid biosynthesis (32). In LPAAT3-KO mice, DHA-containing phospholipids, but not other PUFA-containing species, in the testes were dramatically decreased, suggesting the amount of DHA in the cell membrane is specifically determined by LPAAT3 (supplemental Fig. S5).

The drastic and specific decrease of DHA-containing phospholipids in LPAAT3-KO mice led to male infertility due to the failure in spermiogenesis. It is known that mammalian sperm membranes have a high amount of PUFA-containing phospholipids, not only DHA but also other PUFAs such as eicosapentaenoic acid and arachidonic acid (19). So far, a large proportion of PUFA in the sperm cell membrane is considered important for critical events during membrane fusion, such as acrosome formation and fusion with eggs. This is thought to be due to the highly fluid properties of PUFA-enriched membranes (33). For instance, fatty acid desaturase 2 (FADS2) KO mice fed the AIN93G diet, which lacks all PUFAs except for linoleic acid (18:2, ω-6) and α-linolenic acid (18:3, ω-3), failed to produce mature spermatids as a result of a defect in acrosome formation (18, 34, 35). However, unlike FADS2 KO mice, elongated spermatids with acrosomes were observed in LPAAT3-KO mice (Fig. 6*D*). This difference might derive from the types of decreased PUFA species. Almost all highly unsaturated fatty acid species (except for mead acid, 20:3) are reduced in FADS2 KO mice, whereas only DHA-containing phospholipids are eliminated in LPAAT3-KO mice. In LPAAT3-KO mice, membrane properties required for acrosome formation are likely provided by a compensatory increase in other PUFA-containing species (supplemental Fig. S5, *A* and *B*).

DHA-containing phospholipids increase the ability of cell membranes to deform and vesiculate by its flexible property, which promotes rapid endocytosis (31). Combined with this, we propose that LPAAT3-KO sperm had a defect in the elimination of excess cytoplasm in TBC, which requires the clathrin-mediated endocytosis. Because DHA is more flexible than ω-6 DPA (22:5) due to the additional double bond (36), this would explain how the increased DPA-containing species in LPAAT3-KO failed to compensate for the defects observed in sperm. Conversely, the accumulation of saturated and *trans*-fatty acids, which rigidify the membrane (4, 5), likely impede sperm production. Indeed, diets rich in saturated or *trans*-fatty acids are reported to decrease the quantity and quality of sperm (37, 38). Because Western food is abundant in these fatty acids, and contains little ω-3 fatty acids such as DHA (39), our findings suggest the possible link between metabolic disorders and the reduced male fertility that has been observed in recent decades (40). In addition to their impact on the physical properties of cell membranes, DHA-containing phospholipids might also act as a platform for the functions and trafficking of various proteins needed for proper sperm release and/or for metabolic precursors of DHA-derived lipid mediators, termed docosanoids (12).

In conclusion, by analyzing LPAAT3-KO mice, we identified the novel and specific function of DHA for spermiogenesis. In addition, although the altered DHA levels are associated with several pathological conditions, including obesity, cancer, and aging (41–44), the causal relationship between DHA deficiency and diseases is largely unknown. Therefore, the LPAAT3-KO mice provide a useful and timely tool for studying the roles of DHA *in vivo*.

## Experimental procedures

### Animals

All animal experiments were approved and performed in accordance with the guidelines of the Animal Research Committee of the National Center for Global Health and Medicine (12053, 13009, 14045, 15037, and 16062), and the animal experimentation committee (P11-045) of the University of Tokyo, in addition to the Biomedical Research Ethics Committee of the Graduate School of Medicine at Chiba University (Approval No. A28-18). All experiments of gene recombination were approved by and performed in accordance with the guidelines of the Biosafety Committee of National Center for Global Health and Medicine (27-P-044).

### KO mice and genotyping

LPAAT3-KO mice were generated from C57BL/6N mouse embryonic stem cells (Agpat3^tm1(EUCOMM)Wtsi^) purchased from the European Conditional Mouse Mutagenesis Project. In this construct, a LacZ cassette with a splice acceptor is transcribed from the endogenous promoter in the form of a fusion transcript in which exon1–3 spliced to the *lacZ* gene (supplemental Fig. S3*A*). Mice were genotyped by PCR using primers annealing to the genomic regions indicated in supplemental Fig. S3*A* (WT forward, accttatgaaggtgaccatgtggag; KO forward, cgtcgagaagttcctattccgaagt; Common reverse, gtgtcctgaatgaccaggaagagaa). The expected product sizes are 381 bp for the WT allele and 244 bp for the KO allele.

### Immunohistochemistry

Mouse testes were fixed with Bouin's solution by perfusion through the left ventricle and then immersed in Bouin's solution at room temperature for 1 h. Samples were then embedded in paraffin and sectioned at a thickness of 4 μm. Sections were deparaffinized with xylene and autoclaved in Instant Antigen Retrieval Solution H (LSI Medience Corp., Tokyo, Japan) at 121 °C for 5 min. Samples were then permeabilized with 0.1% Triton X-100/PBS for 15 min, blocked in the blocking solution (5% normal goat serum and 3% bovine serum albumin (BSA) (Sigma) in PBS) for 30 min, and incubated with anti-LPAAT3 antibody (7.07 μg/ml) (24) in blocking solution overnight at 4 °C. Sections were incubated with anti-rabbit IgG antibody conjugated to Alexa 555 (1:2000) (Thermo Fisher Scientific, Waltham, MA), *Arachis hypogaea* agglutinin (PNA)-FITC (1:2000, J-Oil Mills, INC., Tokyo, Japan), and Hoechst 33258 (1:300) (Sigma) to stain the acrosome and nucleus.

### Preparation of testicular single-cell suspensions

The testes albuginea were removed, and the seminiferous tubules were settled in PBS for several minutes. Samples were dissociated by enzymatic digestion with collagenase type I (Sigma) (1 mg/ml) for 10 min at 37 °C in PBS. Samples were then centrifuged at 340 × *g* at 4 °C for 5 min to pellet the seminiferous tubules. The pellets were treated with trypsin (0.25%) and DNase I (0.5 units; Invitrogen) for 10 min at 37 °C. After neutralizing with DMEM (Nacalai Tesque, Kyoto, Japan), 10% fetal bovine serum (FBS) (Thermo Fisher Scientific) was added, and cell suspensions were passed through 40-μm cell strainers (Thermo Fisher Scientific). Samples were centrifuged at 340 × *g* at 4 °C. Testicular cells were collected (1 × 10^6^ cells/ml) and stained with Hoechst 33342 (2.5 μg/ml; Invitrogen) for 90 min at 37 °C. Cells were then washed twice with 2% FBS/PBS and resuspended in propidium iodide (1 μg/ml) in 2% PBS.

### Flow cytometric analysis and cell sorting

Single-cell suspensions of adult mouse testes were analyzed and sorted using a SORP FACSAria (BD Biosciences). Propidium iodide-stained cells were discriminated as dead cells. Testicular cells were classified into three major populations (haploid, diploid, and tetraploid) based on the fluorescence intensity of Hoechst 33342. Subpopulations were determined and gated by their forward and side scatter properties according to previous studies (45). Gating is shown in supplemental Fig. S2.

### Gene expression analysis

Total RNA was isolated from 10,000 germ cells (spermatogonia, spermatocyte, and RS) using an RNeasy mini kit (Qiagen, Hilden, Germany). Total RNA from 10,000 cells was used as a template for complementary DNA synthesis. SuperScript III reverse transcriptase and random primers (Thermo Fisher Scientific) were used for reverse transcription. Gene expression was quantified using the Fast SYBR Green Master Mix with the Step One Plus real-time PCR system (Applied Biosystems). Relative mRNA levels were calculated using the comparative cycle threshold method and normalized to glyceraldehyde-3-phosphate dehydrogenase (*GAPDH*) mRNA. The maximum mRNA expression values for each group were represented as one. The primer sequences used for quantitative PCR and the resulting amplicon sizes were as follows: *GAPDH* (188 bp), forward 5′-tgacaatgaatacggctacagca-3′ and reverse 5′-ctcctgttattatgggggtctgg-3′; *LPAAT3* (174 bp), forward 5′-acctataccgccgtatcaactgc-3′ and reverse 5′-agtcgatctcgaagttgtggttg-3′; *Sperizin* (156 bp), forward 5′-aaccactcactggacccactagt-3′ and reverse 5′-tacggtcacctccaagtcctcaa-3′; *DMRT1* (191 bp), forward 5′-gaccccgcctactacagca-3′ and reverse 5′-gtctgagcaggcacgtaagg-3′; and *TNP1* (120 bp), forward 5′-accagccgcaagctaaagac-3′ and reverse 5′-tttcctacttttcaggacgctc-3′.

### Sperm suspension

The cauda epididymis was removed from sacrificed male mice. The duct of the epididymis was cut, and sperm were collected by gently squeezing the epididymis. Collected sperm were dropped into HTF medium (ARK Resource, Kumamoto). For insemination, sperm were allowed to capacitate in an incubator (37 °C, 5% CO_2_) for 60 min.

### IVF

For inducing superovulation, 7.5 IU of equine chorionic gonadotropin (ASUKA Pharmaceutical, Tokyo, Japan) was intraperitoneally injected into C57BL/6N mature female mice, followed by intraperitoneal injection of 7.5 IU of human chorionic gonadotropin (ASUKA Pharmaceutical) after 48 h. At 15 h after injection, mice were euthanized. Oviducts were dissected and immersed in 200 μl of HTF medium in a fertilization dish. The ampulla of the oviducts was cut, and 4–5 cumulus-oocyte complexes (COC) were obtained. These were added to the HTF medium and covered with mineral oil (Sigma). The fertilization dish was incubated at 37 °C, 5% CO_2_ in air for 30–60 min. 2 μl of sperm suspension was added (300 to 500 sperm/μl) into the center of COC-containing HTF medium. The fertilization dish was incubated at 37 °C, 5% CO_2_ in air for 5 h. Oocytes were then washed three times in fresh HTF medium in a washing dish. At 24 h after insemination, fertilization rates were calculated as the total number of two-cell stage embryos divided by the total number of inseminated oocytes multiplied by 100%.

### Sperm–oocyte fusion assay

The COC obtained from superovulated female mice described previously were treated with hyaluronidase (0.5 mg/ml) (Sigma) to remove cumulus cells. Oocytes were then treated with Tyrode's solution Acidified (Irvine Scientific, Santa Ana, CA) and incubated in potassium simplex optimized medium (KSOM) (ARK Resource) to remove the zona pellucida. Zona pellucida-free oocytes were incubated with sperm. After incubation for 6 h at 37 °C, binucleated oocytes were identified as sperm-fused eggs. The fusion rate was calculated as the total number of binucleated oocytes divided by the total number of inseminated oocytes multiplied by 100%.

### Measurement of male reproductive hormones

Levels of serum testosterone, LH, and FSH were quantified by using enzyme immunoassay kits: Testosterone Parameter Assay kit KGE010 (R&D Systems, Minneapolis, MN), ELISA kit for LH CEA441Mu (Cloud-Clone Corp., Houston, TX), and ELISA kit for FSH CEA830Mu (Cloud-Clone Corp.).

### Detection of apoptotic cells

For the detection of apoptotic cells in the testes, end-labeled by terminal deoxynucleotidyltransferase dUTP nick-end labeling (TUNEL) staining was used. Apoptotic germ cells on paraffin-embedded testis tissue sections were detected using the *in situ* apoptosis detection kit (Takara Biochemicals, Shiga, Japan).

### Tissue homogenate preparation

Mouse testes were homogenized in TSC buffer (100 mm Tris-HCl, pH 7.4, 300 mm sucrose with a complete protease inhibitor mixture) (Roche Applied Science, Basel, Switzerland). After removing the tissue debris (800 × *g*, 10 min, 4 °C) and crude mitochondrial fractions (9,000 × *g*, 10 min, 4 °C), microsomal fractions were collected by ultracentrifugation (100,000 × *g*, 60 min, 4 °C). The pellets (microsomal fractions) were resuspended in ice-cold TSE buffer (20 mm Tris-HCl, pH 7.4, 1 mm EDTA, 300 mm sucrose). Protein concentrations were quantified using the Pierce BCA Protein Assay kit (Thermo Fisher Scientific).

### Western blotting

Testicular microsomal or cytosolic proteins were resolved by 10% SDS-PAGE and transferred to a Hybond ECL nitrocellulose membrane (GE Healthcare, Little Chalfont, UK). The membrane was blocked with blocking buffer (5% skim milk in Tris-based buffer with 0.1% Tween 20) at 4 °C for 16 h. Anti-mouse LPAAT3 antibody (24), anti-mouse nectin-2 antibody (ab135246; Abcam, Cambridge, UK), anti-MMP2 antibody (ab37150; Abcam), and MT1-MMP (ab73879; Abcam) were used in this study. The membrane was incubated with primary antibody at room temperature for 60 min, washed three times for 5 min each with wash buffer (Tris-based buffer with 0.1% Tween 20), and then incubated with an anti-rabbit IgG antibody conjugated to horseradish peroxidase (GE Healthcare) at room temperature for 60 min. The membrane was washed three times for 5 min each with wash buffer. The membrane was developed with ECL reagent (GE Healthcare), and immunoreactive proteins were visualized using the ImageQuant LAS 500 (GE Healthcare).

### Immunocytochemical staining of sperm

Sperm suspensions were washed with PBS two times, fixed with 4% paraformaldehyde, and dried on glass slides. Fixed sperm were then permeabilized with 0.5% Triton X-100 on ice for 10 min, blocked in normal goat serum in PBS at room temperature for 30 min, and then incubated with an anti-ubiquitin antibody (U5379; Sigma) at a dilution of 1:100. Sperm were then incubated with a secondary antibody, and the detection of signal was performed using the VECTASTAIN Elite ABC kit (Vector Laboratories, Cambridgeshire, UK). 3,3′-Diaminobenzidine (Vector Laboratories) was used for colorimetric development.

### LPAAT assay

The microsomal fractions (1 μg/tube) from the mouse testes were incubated with 50 μm deuterium-labeled C16:0 LPA (*d*_13_-LPA, Avanti Polar Lipids, Alabaster, AL) and 5 μm each of 16:0-, 18:1-, 20:4-, and 22:6-CoA (Avanti Polar Lipids) in TSE buffer in a total volume of 100 μl for 10 min at 37 °C. Enzymatic reactions were stopped by adding 300 μl of chloroform/methanol (1:3, v/v) containing the 1.38 μm of di-myristoyl-PA (internal standard, Avanti Polar Lipids). Lipids were extracted using the method of Bligh and Dyer (46), and dried using a centrifugal evaporator (Sakuma Seisakusho Ltd., Tokyo, Japan). The dried lipids were reconstituted in 100 μl of methanol. Products were separated on an AQUITY ultra-performance liquid chromatograph (UPLC) BEH amide column (1.7 μm, 2.1 × 30 mm) with a linear gradient of mobile phase B (acetonitrile) over the mobile phase A (20 mm NH_4_HCO_3_/water) using an AQUITY UPLC system (Waters) at a flow rate of 800 μl/min. The elution gradient was as follows (% A/% B): 0 min (5:95) to 5 min (80:20) to 10 min (80:20) to 15 min (5:95). The reaction products and the internal standard were analyzed using a TSQ Vantage triple stage quadrupole mass spectrometer (Thermo Fisher Scientific) by selected reaction monitoring (SRM). Each PA species was detected by the combination of [M − H]^−^ at Q1 and [fatty acid (FA) − H]^−^ at Q3. The amount of LPAAT assay product was calculated using the peak area value. Finally, LPAAT activities were represented as a normalized area value by internal standard.

### Lipid extraction for phospholipid composition analysis

Tissues or cells were homogenized in TSC buffer or buffer RLT from the RNeasy mini kit (Qiagen). The tissue microsomal fraction or tissue/cell lysate (800 × *g* supernatant) was used for the lipid extraction. For PC, PE, and PS analyses, lipids were extracted using the method of Bligh and Dyer (46). Subsequently, lipids were dried using a centrifugal evaporator and reconstituted in methanol. For the PA analysis, acidic lipids were concentrated using a diethylaminoethylcellulose (DEAE-cellulose) column, as described previously (47). Briefly, 50 μl of tissue homogenate (800 × *g* supernatant) was mixed with 50 μl of di-myristoyl-PA (1 μm in methanol), 750 μl of *n*-butanol, 30 μl of phosphate buffer (500 mm, pH 5.8), and 170 μl of water. The samples were then vortexed for 5 min, sonicated for 3 min in a bath sonicator, and centrifuged at 1000 × *g* for 5 min at 4 °C. An upper layer (700 μl) consisting of the first butanol extract was transferred to the new tube. For the second extraction, 700 μl of ethyl acetate/hexane (1:1, v/v) was added to the remaining lower layer. After vortexing for 5 min and centrifuging at 1000 × *g* for 5 min at 4 °C, 700 μl of the upper layer (second extract) was combined with an equal volume of the first butanol extract and methanol. The combined lipid extract totaling 1.5 ml was loaded onto the DEAE-cellulose column. The column was washed with 2 ml of methanol, and then the acidic lipids containing PA were extracted with 1 ml of methanol/ammonia/formic acid (1000:33:22, v/v). The extract was dried using a centrifugal evaporator and reconstituted in 120 μl of methanol.

### Analysis of phospholipids

Each phospholipid was measured using the LC-MS/MS using SRM (LC-SRM-MS). For PC, PE, and PS, LC-SRM-MS analyses were performed using a Nexera UHPLC system and triple quadrupole mass spectrometers LCMS-8050 (Shimadzu Corp., Kyoto, Japan) as we reported previously (48). The extracted lipids (5 μl/injection) were separated on an Acquity UPLC BEH C8 column (1.7 μm, 2.1 × 100 mm, Waters) with a gradient of mobile phase A (5 mm NH_4_HCO_3_/water), mobile phase B (acetonitrile), and mobile phase C (isopropyl alcohol) at a flow rate of 0.35 ml/min. The gradient setting was as follows: time (% A)/(% B)/(% C): 0 min (75:20:5) to 20 min (20:75:5) to 40 min (20:5:75) to 45 min (5:5:90) to 50 min (5:5:90) to 55 min (75:20:5). The column oven temperature was set to 47 °C. SRM analysis of each phospholipid species was performed with the following transitions (Q1 and Q3): ([M + H]^+^, 184) for PC, ([M + H]^+^, neutral loss of 141) for PE, and ([M − H]^−^, neutral loss of 87) for PS. Additional SRM analyses with detection of fatty acid fragments at Q3 were performed using the testicular lipid extracts. SRM analyses were performed as follows (Q1 and Q3): ([M + HCO_3_]^−^, [FA − H]^−^) for PC and ([M − H]^−^, [FA − H]^−^) for PE. In this analysis, Q3 channels were set for the following fatty acids: 14:0, 14:1, 16:0, 16:1, 18:0 to 18:3, 20:0 to 20:4, 22:0 to 22:6, 24:0 to 24:6. The obtained retention time of each species was used for the peak identification of DHA-containing species in testes and germ cells (Fig. 2, *C–F*). For PA, LC-SRM-MS analysis was performed using an AQUITY UPLC system and TSQ Vantage triple stage quadrupole mass spectrometer (47). The extracted lipids (5 μl/injection) were separated on an Acclaim PepMap 100 C18 column (3 μm, 150 × 1.0-mm inner diameter, Thermo Fisher Scientific) with a linear gradient of mobile phase A (isopropyl alcohol/methanol/water, 5:1:4) and mobile phase B (isopropyl alcohol). Both mobile phases also contained 0.2% formic acid, 0.028% ammonia, and 5 μm phosphoric acid. The gradient transition was as follows (% A/% B): 0 min (70:30) to 2 min (50:50) to 12 min (30:70) to 12.5 min (5:95) to 22.5 min (5:95) to 23 min (95:5) to 24 min (95:5) to 26 min (70:30). SRM analysis of each PA species was performed with the following transitions (Q1 and Q3): ([M + NH_4_]^+^, neutral loss of 115). In the case of PA, 5 μl of the mobile phase A was injected after every sample to determine the background of each experiment.

### Quantification of phospholipids

Deisotoping was performed manually using the retention time and signal intensity. For PA, the obtained background peak area values (see under “Analysis of phospholipids”) were subtracted. If two or more peaks were seen in the single SRM channel, all peaks areas were combined, except for the cases when fatty acid combination at the *sn*-1 and *sn*-2 positions was determined, and each peak area value was determined separately (Fig. 2, *C–F*). To calculate the fatty acid composition of phospholipids, peak areas of individual species were normalized with the sum of all the detected species and shown as a percentage of the total species. Phospholipid species in excess of 1% of the total species are shown as figures.

### Annotation of phospholipids

Each phospholipid species in the LC-SRM-MS analyses was denoted as *X*-AA:B. *X* indicates the name of each phospholipid, whereas AA and B are the sum of the number of carbons and double bonds in fatty acids at *sn*-1 and *sn*-2 position, respectively. In the case of PLs with ether bonds (alkyl species) and vinyl ether bonds (plasmalogen), the molecule is denoted as o*X*-AA:B and p*X*-AA:B, respectively. When different species displayed the same *m/z* values, *e.g.* oPC-34:1 and pPC-34:0, they are denoted as oPC-34:1/ePC-34:0. If the fatty acid combinations at *sn*-1 and *sn*-2 were identified, phospholipid species was denoted as *X*-CC:D/EE:F. In this case, CC (and EE) is the number of carbons, and D (and F) is the number of double bonds in fatty acids. These methods do not discriminate which *sn* position harbors each fatty acid residue.

### Scanning electron microscopy

Sperm were fixed in 2.5% glutaraldehyde in PBS and post-fixed with 1% OsO_4_. After fixation, sperm were dehydrated with serially increasing concentrations of ethanol (50–100%). They were incubated with a mixed solution of ethanol and isoamyl acetate and then equilibrated with 100% isoamyl acetate before critical point drying with a JCPD-5 (JEOL, Tokyo, Japan). Samples were placed on specimen block. The conductive coating with platinum was performed using a precision etching coating system Model 682 (Gatan, CA). Imaging was carried out on a JSM-7001F electron microscope (JEOL).

### Low vacuum SEM

Sperm collected from the cauda epididymis were fixed with 2% glutaraldehyde for 2 h. Samples were washed three times with PBS, air-dried, and coated for 30 min at room temperature with a platinum blue solution, which was adjusted to pH 9 by adding a small volume of ammonia (TI-blue staining kit, Nisshin Em Co., Ltd., Tokyo, Japan). After washing with distilled water for 1–2 min, SEM images were obtained using a low SEM microscope Miniscope® TM3030 (Hitachi, Tokyo, Japan).

### Transmitted electron microscopy

Adult testes were fixed with 2.5% glutaraldehyde in 0.1 m phosphate buffer by perfusion through the left ventricle. Samples were then immersed in the same fixative overnight at 4 °C and then post-fixed with 2% osmium tetroxide. Samples were dehydrated through a graded ethanol series and embedded in Epon 812 (TAAB Laboratories Equipment, Berks, UK). Sections 1 μm in thickness were cut on an ultramicrotome (Ultracut E; Reichert-Jung, Vienna, Austria) and stained with 1% toluidine blue (Wajdeck GmbH & Co., Munster, Germany). Ultrathin sections were also cut and stained with uranyl acetate and lead citrate. The sections stained with toluidine blue were visualized on an Olympus BX50 light microscope and analyzed using images captured by a CCD camera RETIGA Exi FAST 1394 (Qimaging, Surrey, Canada). The ultrathin sections were observed using a JEOL JEM-1200 EX TEM (JEOL).

### Liposome preparation

Liposomes were prepared by obtaining 500 nmol of palmitoyloleoyl-PC (POPC, PC-16:0/18:1), palmitoylarachidonoyl-PC (PAPC, PC-16:0/20:4), and palmitoyldocosahexaenoyl-PC (PDPC, PC-16:0/22:6) in chloroform. All lipids were obtained from Avanti Polar Lipids. These samples were dried under nitrogen gas in a glass tube. The lipids were dried further using a centrifugal evaporator for 15 min at 37 °C. The lipid films were reconstituted in 500 μl of Tris-NaCl-KCl buffer (TNK; 12.38 mm Tris-HCl, pH 7.5, 68.44 mm NaCl, 1.34 mm KCl). After vortexing for 1 min, the samples were subjected to five freeze-thaw cycles using liquid nitrogen and a 37 °C water bath. Finally, the liposomes were passed through a polycarbonate filter (inner diameter 200 nm, Whatman) 21 times using a mini-extruder (Avanti Polar Lipids).

### Liposome filter passage index

To monitor the pressure change caused by liposomes passing through the filter, a polycarbonate filter (30 nm pore size) was connected to the ÄKTA explorer chromatography system (ÄKTA explorer 10S, GE Healthcare). The assays were performed at 25 °C. TNK buffer was used as a mobile phase at a flow rate of 1 ml/min. First, 100 μl of TNK buffer was injected to measure the reference pressure for 2 min. Next, a 100-μl solution containing the 200-nm sized liposomes (1 mm, prepared as described above) was injected at a flow rate of 1 ml/min for 2 min. The pressure change was obtained by subtracting the reference pressure. The decreased pressure value (maximum pressure) − (pressure at 2 min after injection) was used to evaluate the ease of liposome passage. The liposome filter passage index was calculated as follows: (decreased pressure value)/(maximum pressure value) × 100.

### Statistical analysis

Unpaired *t* tests were used to compare two groups. Multiple comparisons were performed with Bonferroni's multiple comparison tests depending on the combinations of comparisons, after one- or two-way ANOVA. All analyses were done with GraphPad Prism 5 for Mac OS X software (GraphPad Software, Inc., La Jolla, CA).

## Author contributions

Y. I.-H. and D. H. designed the project, performed the experiments, interpreted the data, and wrote the manuscript. J. S. generated the KO mouse. K. Takubo, M. G., K. N., T. O., C. I., and K. Toshimori performed the experiments and interpreted the data. H. N. performed the unpublished essential experiments. K. Takubo, H. S., C. I., K. Toshimori, T. Sasaki, and T. Shimizu revised the manuscript. D. H. and T. Shimizu supervised the project. All authors reviewed the results and approved the final version of the manuscript.

## Supplementary Material

Supplemental Data

## Footnotes

4 The abbreviations used are:

DHA: docosahexaenoic acid
RS: round spermatid
PA: phosphatidic acid
PC: phosphatidylcholine
PE: phosphatidylethanolamine
PS: phosphatidylserine
HZ: heterozygote
DPA: docosapentaenoic acid
LH: luteinizing hormone
FSH: follicle-stimulating hormone
IVF: *in vitro* fertilization
aES: apical ectoplasmic specialization
TBC: tubulobulbar complex
MMP: matrix metalloproteinases
MT1-MMP: membrane type-1-MMP
TEM: transmitted electron microscopy
HTF: human tubal fluid
COC: cumulus-oocyte complex
FBS: fetal bovine serum
TSC: Tris sucrose with a complete protease inhibitor cocktail
TSE: Tris sucrose EDTA
SRM: selected reaction monitoring
LPA: lysophosphatidic acid
UPLC: ultra-performance liquid chromatography
POPC: palmitoyloleoyl-PC
PAPC: palmitoylarachidonoyl-PC
PDPC: palmitoyldocosahexaenoyl-PC
ANOVA: analysis of variance
LPAAT: LPAAT, lysophosphatidic acid acyltransferase.
