# Supplementary material for: Lysophosphatidic acid acyltransferase 3 tunes the membrane status of germ cells by incorporating docosahexaenoic acid during spermatogenesis
Source: J Biol Chem. 2017 Jun 3;292(29):12065–76. doi: 10.1074/jbc.M117.791277 (PMC5519358; doi:10.1074/jbc.M117.791277)
Supplement: Supplemental Data [file supp_292_29_12065__index.html]

Lysophosphatidic acid acyltransferase 3 tunes the membrane status of germ cells by incorporating docosahexaenoic acid during spermatogenesis. — Lysophosphatidic acid acyltransferase 3 tunes the membrane status of germ cells by incorporating docosahexaenoic acid during spermatogenesis — LPAAT3 produces DHA-enriched membrane for spermatogenesis — Supplemental Data 

# Lysophosphatidic acid acyltransferase 3 tunes the membrane status of germ cells by incorporating docosahexaenoic acid during spermatogenesis

## Supplemental Data

- Suplemental Figure S1 (.jpg, 1.1 MB) - Diagram of 12-stage cycles of spermatogenesis in mice.
- Suplemental Figure S2 (.jpg, 1.1 MB) - Sorting of mouse germ cells.
- Suplemental Figure S3 (.jpg, 774 KB) - Generation of LPAAT3 KO mice.
- Suplemental Figure S4 (.jpg, 1.1 MB) - Phospholipid profiles in testes.
- Suplemental Figure S5 (.jpg, 1.8 MB) - Phospholipid profiles in germ cells.
- Suplemental Figure S6 (.jpg, 692 KB) - Schematic of liposome filtration assay.
