# Supplementary figures and images for: Lysophosphatidic acid acyltransferase 3 tunes the membrane status of germ cells by incorporating docosahexaenoic acid during spermatogenesis
Source: J Biol Chem. 2017 Jun 3;292(29):12065–76. doi: 10.1074/jbc.M117.791277 (PMC5519358; doi:10.1074/jbc.M117.791277)

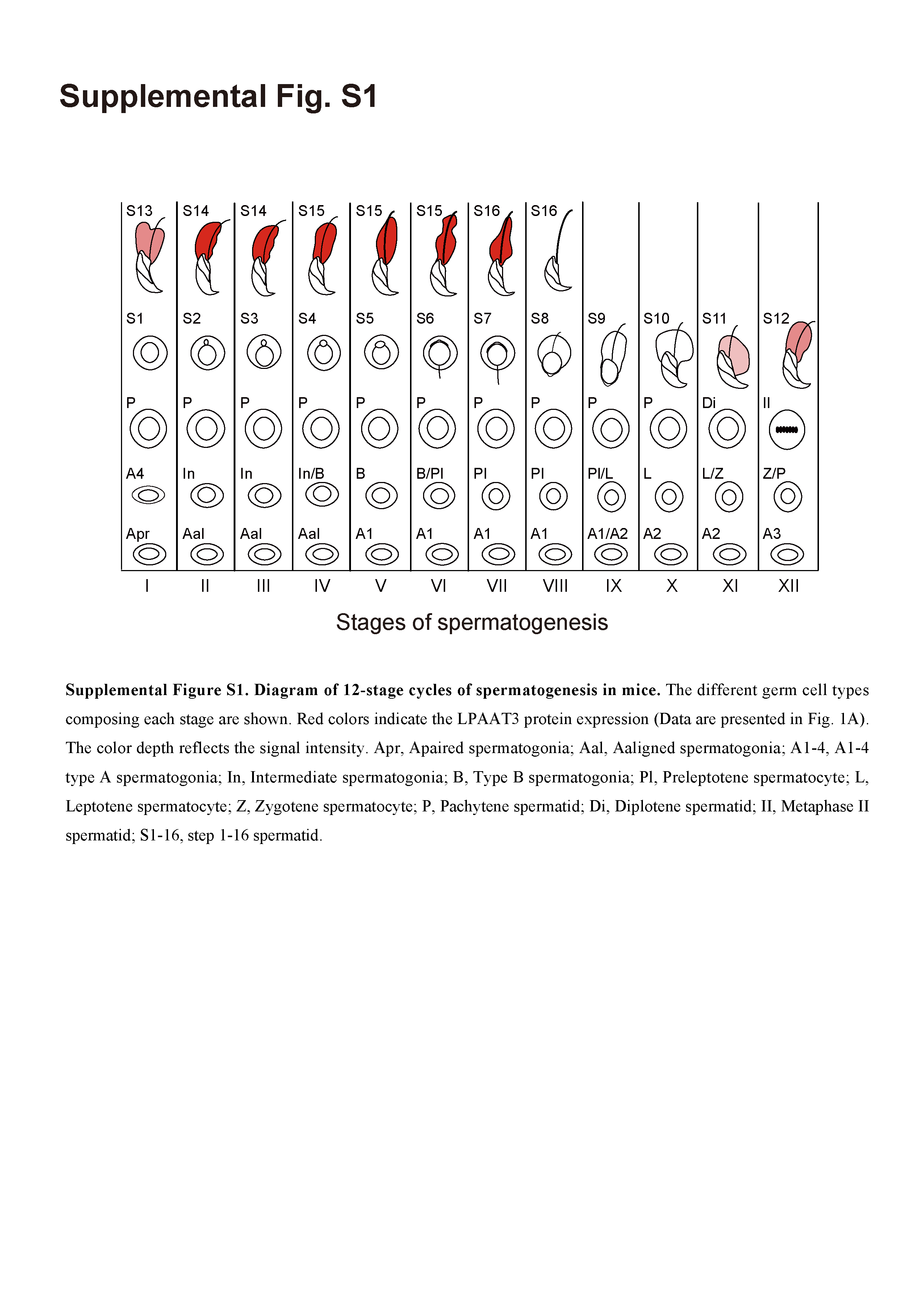

Supplement: Supplemental Data [file 10.1074_M117.791277_jbc.M117.791277-1.jpg]

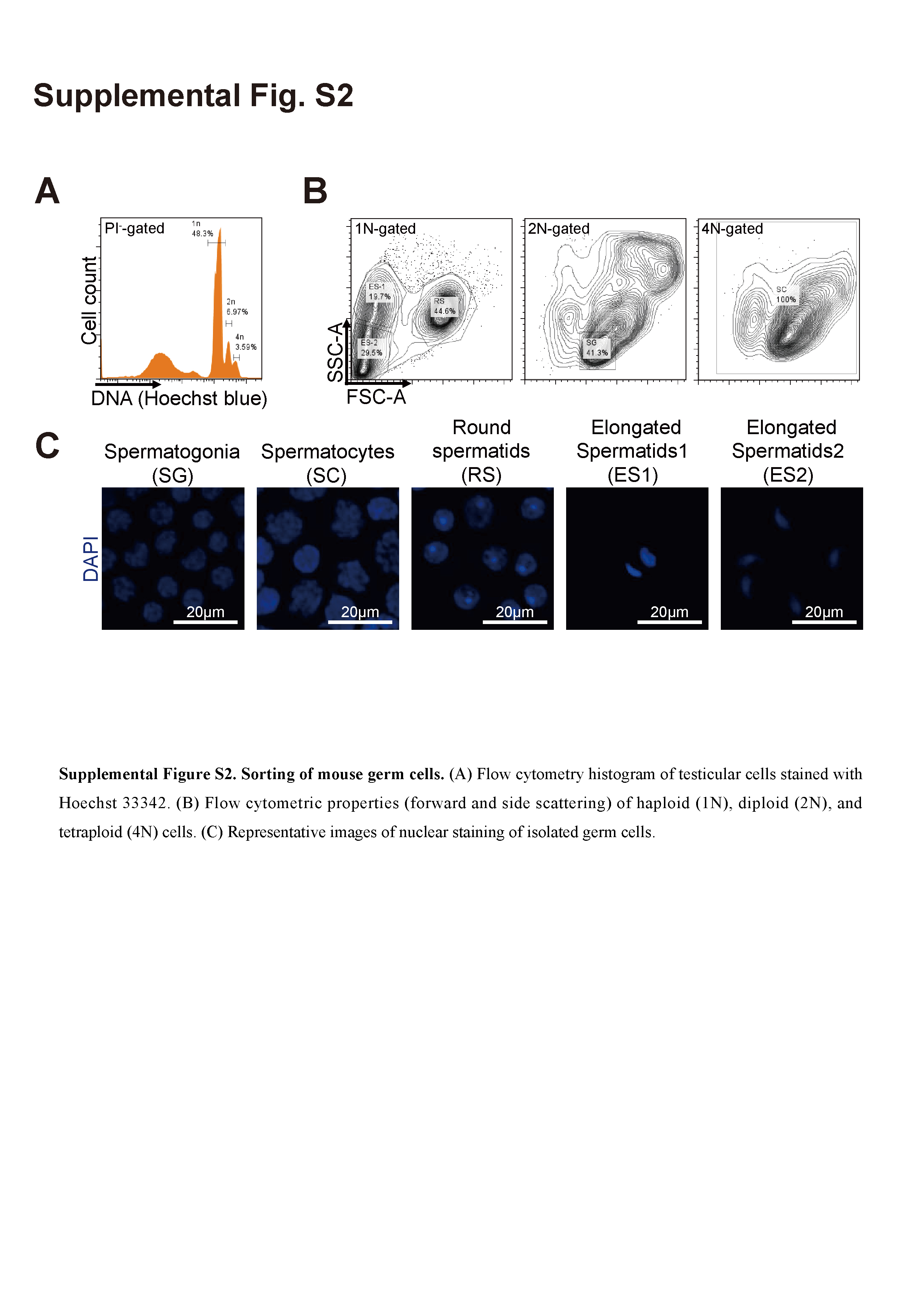

Supplement: Supplemental Data [file 10.1074_M117.791277_jbc.M117.791277-2.jpg]

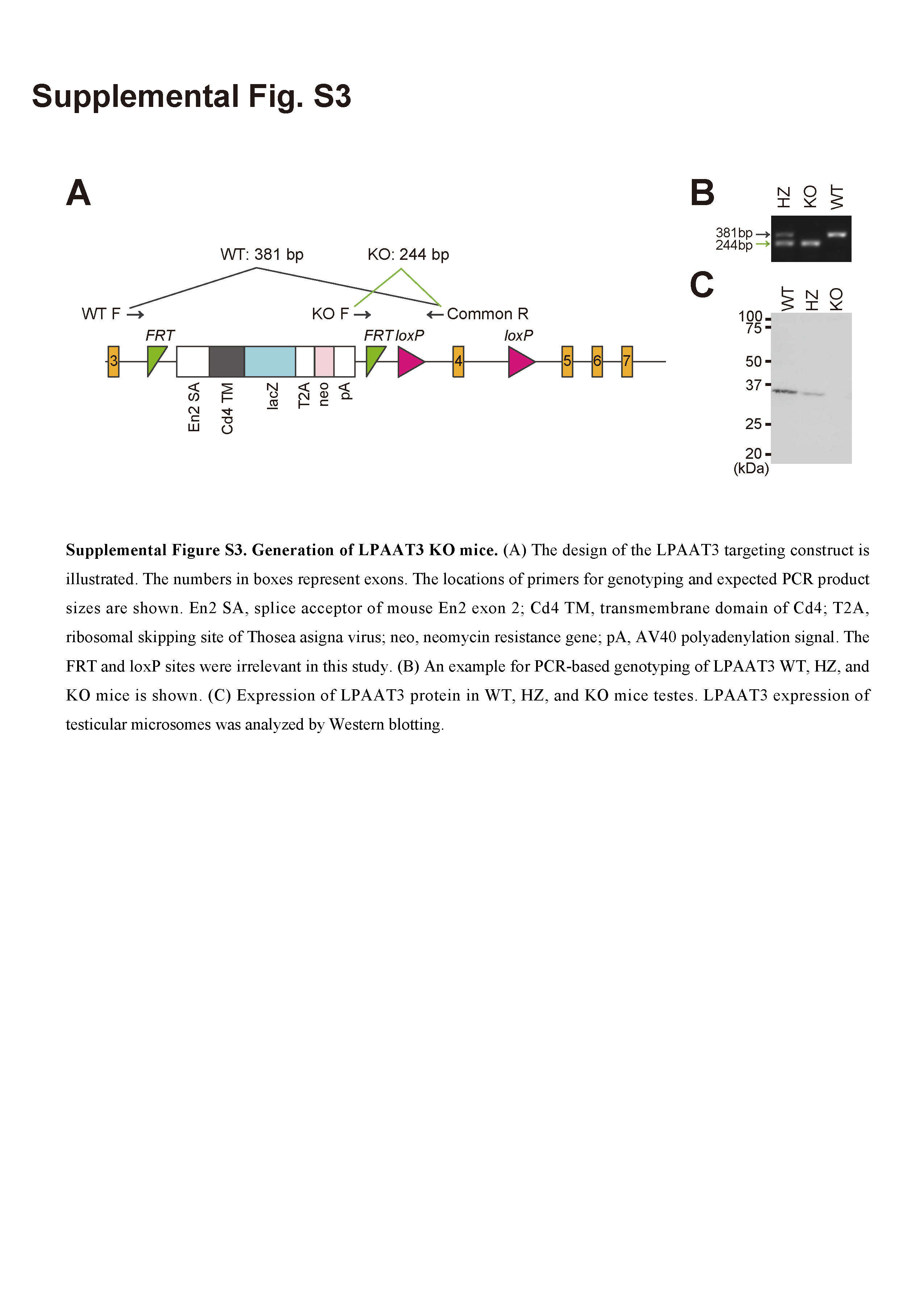

Supplement: Supplemental Data [file 10.1074_M117.791277_jbc.M117.791277-3.jpg]

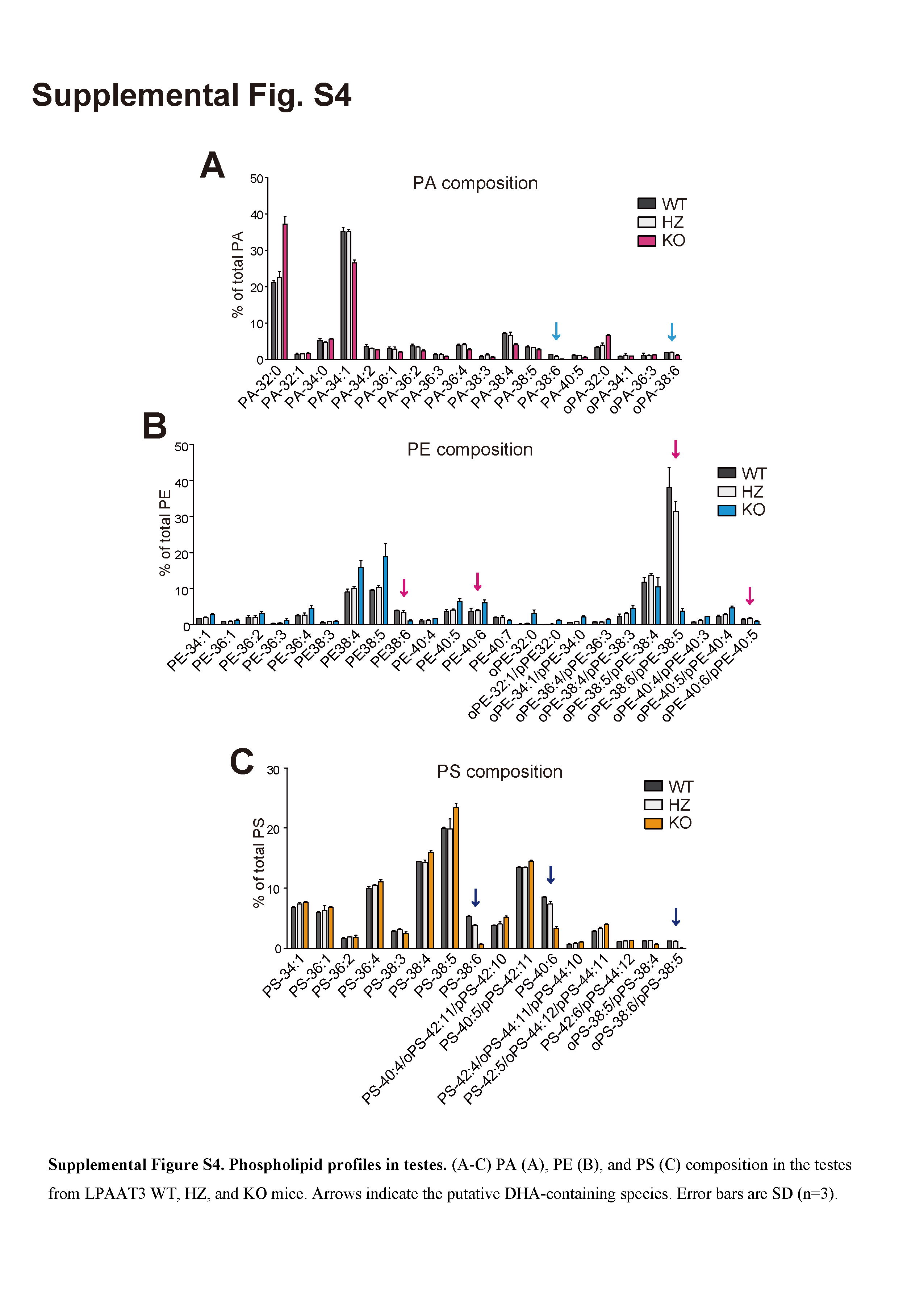

Supplement: Supplemental Data [file 10.1074_M117.791277_jbc.M117.791277-4.jpg]

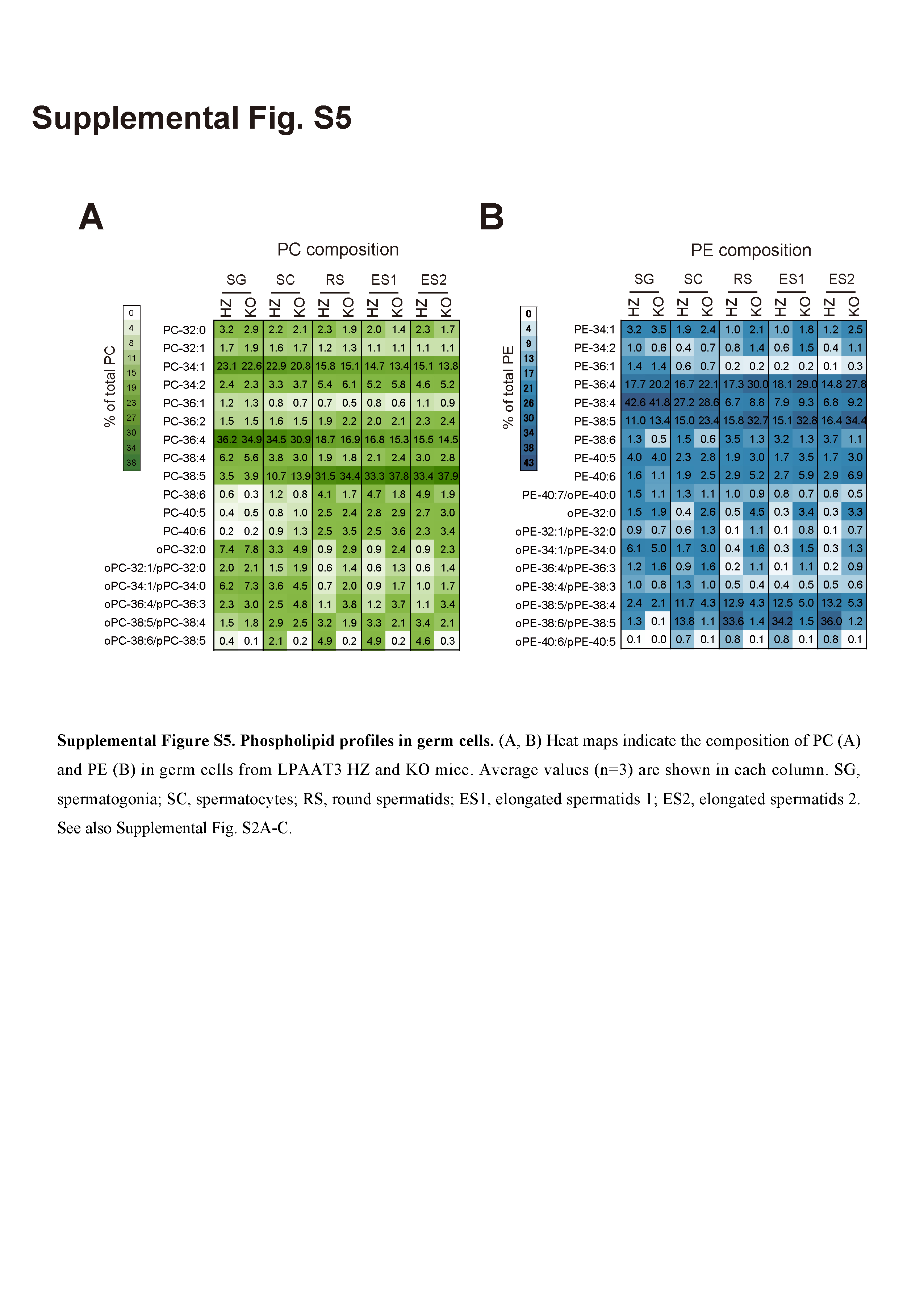

Supplement: Supplemental Data [file 10.1074_M117.791277_jbc.M117.791277-5.jpg]

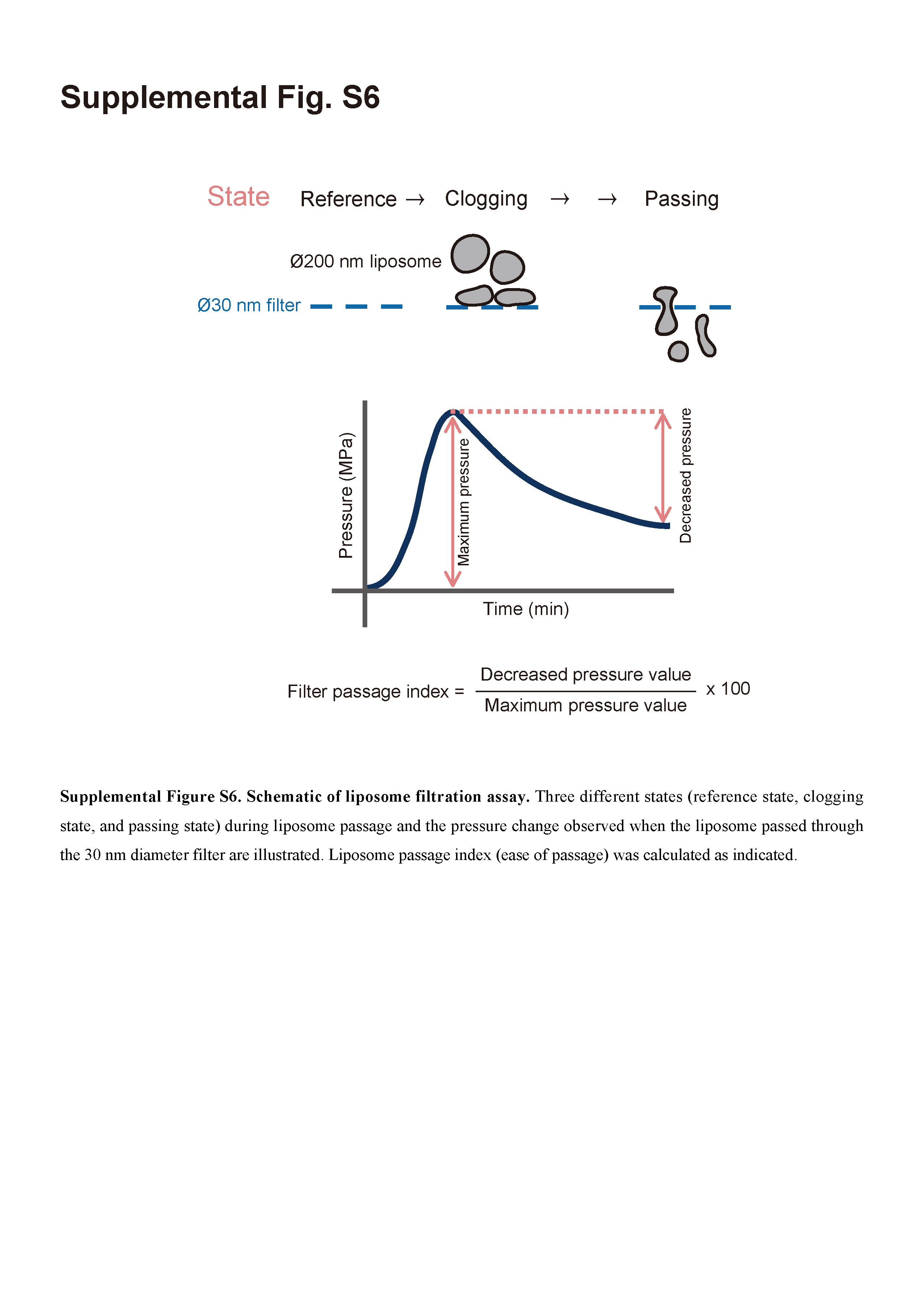

Supplement: Supplemental Data [file 10.1074_M117.791277_jbc.M117.791277-6.jpg]
